# Evaluation of mouse urinary bladder smooth muscle for diurnal differences in contractile properties

**DOI:** 10.3389/fphar.2014.00293

**Published:** 2015-01-09

**Authors:** Rachel S. White, Betsir G. Zemen, Zulqarnain Khan, Jenna R. Montgomery, Gerald M. Herrera, Andrea L. Meredith

**Affiliations:** ^1^Department of Physiology, University of Maryland School of MedicineBaltimore, MD, USA; ^2^Catamount Research & Development Company and Med Associates Inc., St. AlbansVT, USA

**Keywords:** UBSM, BK channel, *Kcnma1*, circadian rhythm, peripheral rhythm, urodynamics, isometric tension, lower urinary tract

## Abstract

Most physiological systems show daily variations in functional output, entrained to the day–night cycle. Humans exhibit a daily rhythm in urinary voiding (micturition), and disruption of this rhythm (nocturia) has significant clinical impact. However, the underlying mechanisms are not well-understood. Recently, a circadian rhythm in micturition was demonstrated in rodents, correlated with functional changes in urodynamics, providing the opportunity to address this issue in an animal model. Smooth muscle cells from mouse bladder have been proposed to express a functional and autonomous circadian clock at the molecular level. In this study, we addressed whether a semi-intact preparation of mouse urinary bladder smooth muscle (UBSM) exhibited measurable differences in contractility between day and night. UBSM tissue strips were harvested at four time points over the diurnal cycle, and spontaneous (phasic) and nerve-evoked contractions were assessed using isometric tension recordings. During the active period (ZT12-24) when micturition frequency is higher in rodents, UBSM strips had no significant differences in maximal- (high K^+^) or nerve-evoked contractions compared to strips harvested from the resting period (ZT0-12). However, a diurnal rhythm in phasic contraction was observed, with higher amplitudes at ZT10. Consistent with the enhanced phasic amplitudes, expression of the BK K^+^ channel, a key suppressor of UBSM excitability, was lower at ZT8. Higher expression of BK at ZT20 was correlated with an enhanced effect of the BK antagonist paxilline (PAX) on phasic amplitude, but PAX had no significant time-of-day dependent effect on phasic frequency or nerve-evoked contractions. Overall, these results identify a diurnal difference for one contractile parameter of bladder muscle. Taken together, the results suggest that autonomous clocks in UBSM make only a limited contribution to the integrated control of diurnal micturition patterns.

## INTRODUCTION

Most physiological systems, including the urinary system, exhibit daily (24-hr) variations in functional output that are entrained to the day–night cycle. Humans exhibit a daily rhythm in urinary voiding (micturition), and nocturia, excessive urination at night, is a persistent disorder affecting >50% of people in some age groups and significantly decreasing quality of life (Ticher et al., 1994; Hetta, 1999; Neveus et al., 1999; Weiss et al., 2008). The circadian variation in urination depends on daily urine production, the physical properties of the bladder, and neural control. Dysfunction in these pathways may contribute to nocturia, but the identification of causal relationships has been limited. The diurnal variation in glomerular filtration rate (GFR) in the kidney is well-documented in humans and animals (Koopman et al., 1985; Zuber et al., 2009), and in some cases, nocturia in humans is associated with a loss of the diurnal variation in GFR (De Guchtenaere et al., 2007). However, not all cases of nocturia are caused by polyuria. Diminished bladder capacity is a major contributor to nocturia and can result from nocturnal detrusor overactivity and neurogenic bladder (Weiss et al., 2008). Few direct comparisons have been made between the physical properties of the bladder during the day and night under controlled conditions (Herrera and Meredith, 2010). Thus the aspects of the lower urinary tract that influence normal circadian micturition patterns, and consequently that contribute to nocturia, are essentially unknown.

Recently, rodents have been found to be an appropriate model for addressing the basis for daily rhythm in micturition. In rodent models, the day–night difference in urine voiding is in part driven by urine production by the kidney, coordinated through hormonal control via aldosterone and vasopressin linked to the circadian clock (Jin et al., 1999; Zuber et al., 2009). Rats and mice demonstrate a circadian rhythm in micturition frequency and volume, correlated with daily changes in functional bladder capacity (Herrera and Meredith, 2010; Negoro et al., 2012). At night, the rodent’s active period, bladder capacity is reduced and micturition frequency is increased compared to day, when rodents sleep. Both renal and micturition rhythms are disrupted by mutations in ‘clock genes’ that abolish circadian rhythms (Zuber et al., 2009; Negoro et al., 2012; Noh et al., 2014).

To dissect the mechanism of circadian rhythms in micturition, the validation of daily changes in urodynamic properties established the bladder as a target for circadian regulation (Herrera and Meredith, 2010). Like many other peripheral tissues in the body, smooth muscle has been shown to possess intrinsic rhythms (Reilly et al., 2008; Paschos and FitzGerald, 2010; Su et al., 2012). Cultured bladder smooth muscle cells show circadian rhythms in gene expression, suggesting there is an autonomous circadian clock at the level of bladder muscle. Daily oscillations have been observed in several transcription factors previously demonstrated to drive the core clock mechanism in SCN and other peripheral tissues (Negoro et al., 2012; Noh et al., 2014). These transcription factors are linked to Cx43 expression in bladder cells, a gap junction channel that regulates bladder storage capacity, as well as other genes associated with smooth muscle contractility (Negoro et al., 2012). These data predict that UBSM possesses robust autonomous rhythmicity, yet no direct evidence demonstrating daily variations in baseline UBSM contractility has been reported to date.

To address this issue, in this study we recorded contractile activity from urinary bladder smooth muscle (UBSM) strips harvested at four time points to identify any differences in spontaneous and evoked contractile amplitudes over the circadian cycle. The expression pattern of the BK K^+^ channel (K_Ca_1.1, *Kcnma1*), a potent regulator of smooth muscle excitability (Meredith et al., 2004) and output of the central circadian clock (Meredith et al., 2006), was also assessed in UBSM, and contractile activity was recorded in the presence of a BK channel blocker to determine whether the diurnal difference in contractility was reduced.

## MATERIAL AND METHODS

### MICE

All procedures involving mice were conducted in accordance with The University of Maryland School of Medicine animal care and use guidelines. C57BL6/J WT mice were group housed on a standard 12:12 h light:dark cycle (LD) until experimental procedures. Time points over the circadian cycle are referred to as zeitgeber time (ZT), denoting time in hours relative to the 24 h cycle. Lights on is defined as ZT0, and lights off is ZT12. Mice were euthanized by inhalation of saturating isoflurane vapors, followed by rapid decapitation.

### ISOLATION OF UBSM AND WESTERN BLOTTING

For Western blots, mouse (3–4 mo) urinary bladders were solubilized in lysis buffer (137 mM NaCl, 1% Triton X-100, 0.5% deoxycholate, 40 mM HEPES, pH 7.4, 1 mM EDTA, pH 7.4, 2 μg/ml aprotinin, 1 μg/ml leupeptin, 2 μg/ml antipain, 10 μg/ml benzamidine, and 0.5 mM phenylmethylsulfonyl fluoride). The insoluble fraction was separated by centrifugation (14,000 *g* for 5 min). 5 μg of soluble supernatant protein was loaded per lane and subjected to SDS-PAGE on a 7.5% acrylamide gel. Proteins were transferred to a nitrocellulose membrane, and membranes were blocked (4% dry non-fat milk, 2% normal goat serum, 10 mM Tris (pH 8), 0.15 M NaCl, and 0.1% Tween 20) for 1-hr. Primary antibodies in blocking solution were incubated overnight at 4°C each of mouse monoclonal α-*Slo* (1 μg/ml L6.60, Neuromab, University of California at Davis, Davis, CA, USA) and mouse monoclonal DM1a α-tubulin (1:10,000, T-9026, Sigma). Membranes were labeled with 1:500 SuperSignal West Dura horseradish peroxidase-conjugated goat α-rabbit and α-mouse secondary antibodies (Pierce), and proteins were visualized by SuperSignal chemiluminescence detection (Pierce). Densitometry of BK band to DM1α anti-tubulin was performed as described previously (Meredith et al., 2006).

### ISOMETRIC TENSION RECORDINGS

After euthanasia, urinary bladders were removed and placed in ice-cold dissection solution composed of (in mM) 80 monosodium glutamate, 55 NaCl, 6 KCl, 10 glucose, 10 HEPES, and 2 MgCl_2_, with pH adjusted to 7.3 with NaOH. The bladder was cut open to expose the urothelial surface and rinsed several times with dissection saline to remove residual traces of urine. The urothelial layer was carefully dissected away from the smooth muscle layer and discarded. Small strips of detrusor (2–3 mm wide and 5–7 mm long) were cut from the bladder wall. Silk threads were attached to each end of the strips, and the strips were transferred to cold (4°C) physiological saline solution (PSS) containing (in mM) 119 NaCl, 4.7 KCl, 24 NaHCO_3_, 1.2 KH_2_PO_4_, 2.5 CaCl_2_, 1.2 MgSO_4_, and 11 glucose and aerated with 95% O_2_–5% CO_2_ to obtain pH 7.4. Each strip was mounted in a tissue bath (15-ml volume) containing aerated PSS (95% O_2_–5% CO_2_, 37°C; MyoMED myograph system; Catamount Research and Development Inc., St. Albans, VT). Initial tension was applied as indicated, and strips were equilibrated for 45 min with bath solution exchanges every 15 min. 60 mM KCl in PSS was delivered for 5 min to produce a maximal contraction, and then washed out with two 10 min PSS washes. KCl-induced contractions were repeated twice. Strips with no baseline contractile activity were not included in the dataset. KCl-induced contractile amplitudes were determined from the third KCl application, either the maximal contractile amplitude (peak) or 5 min post-KCl (steady-state). Area under the curve (AUC) values were obtained from the integral of the contractile response covering the initial rise to 5 min post-KCl. All time points indicate the time of contractile assays.

For phasic contractions, force transducers were calibrated for 1 g and contractile activity was recorded for 30 min after the KCl applications and wash out (Herrera et al., 2003; Meredith et al., 2004). Frequency and amplitude were determined for each strip from 5 min of continuous spontaneous activity within the 30 min recording window (MiniAnalysis, Synaptosoft, Inc.). Phasic amplitude values were normalized to the KCl-evoked amplitude to account for any variability in cutting the strips. AUC and rise time values were obtained from each contractile event in the 5 min period (MiniAnalysis, Synaptosoft, Inc.) and averaged for each strip. For pharmacology experiments, Paxilline (PAX; 10 μM; Sigma) or DMSO (0.1% vehicle control) was added in each chamber after 30 min. Analysis of phasic activity after drug or vehicle was performed on 5 min of continuous spontaneous activity, 30 min after Pax or DMSO application.

For nerve-evoked contractions, frequency-response curves were constructed by measuring the electric field stimulation (EFS)-induced contraction amplitude at stimulus frequencies of 0.5, 2, 3.5, 5, 7.5, 10, 12.5, 15, 20, 30, 40, and 50 Hz. Pulse amplitude was 20–30 V of alternating polarity. Pulse width was 0.2 ms, and stimulus duration was 2 s. Stimuli were given every 3 min using a model PHM-152V stimulator (Catamount Research and Development Inc; Herrera et al., 2005; Werner et al., 2007). Amplitude was determined in Myograph software (Catamount Research and Development, Inc.). EFS-evoked amplitude values were normalized to the KCl-evoked amplitude. EFS-evoked amplitudes normalized to the 50-Hz amplitude value were fit with a standard exponential function to derive the frequency of half maximal activation (OriginLab, Northampton, MA, USA). For pharmacology experiments, one 5 min PSS wash was conducted after the first EFS, followed by addition of Pax (10 μM) or DMSO (0.1%) and a post-drug EFS after 30 min.

### STATISTICS

Group averages are reported ±SE. Reported n’s are the number of animals, with 1–4 strips averaged together for each animal as indicated in figure legends. Statistical significance was determined across time points at *p* < 0.05 by one-way ANOVA (or repeated measures ANOVA across frequencies EFS-evoked contractions across time points) with Bonferroni *post hoc* tests in SPSS v19 (IBM Corp., Armonk, NY, USA). Cosinor analysis was performed with software available at http://www.circadian.org/software.html (Refinetti et al., 2007).

## RESULTS

### BASELINE AND PHASIC CONTRACTILE ACTIVITY IN MOUSE UBSM AT DIFFERENT TIMES OF DAY

**FIGURE 1:**
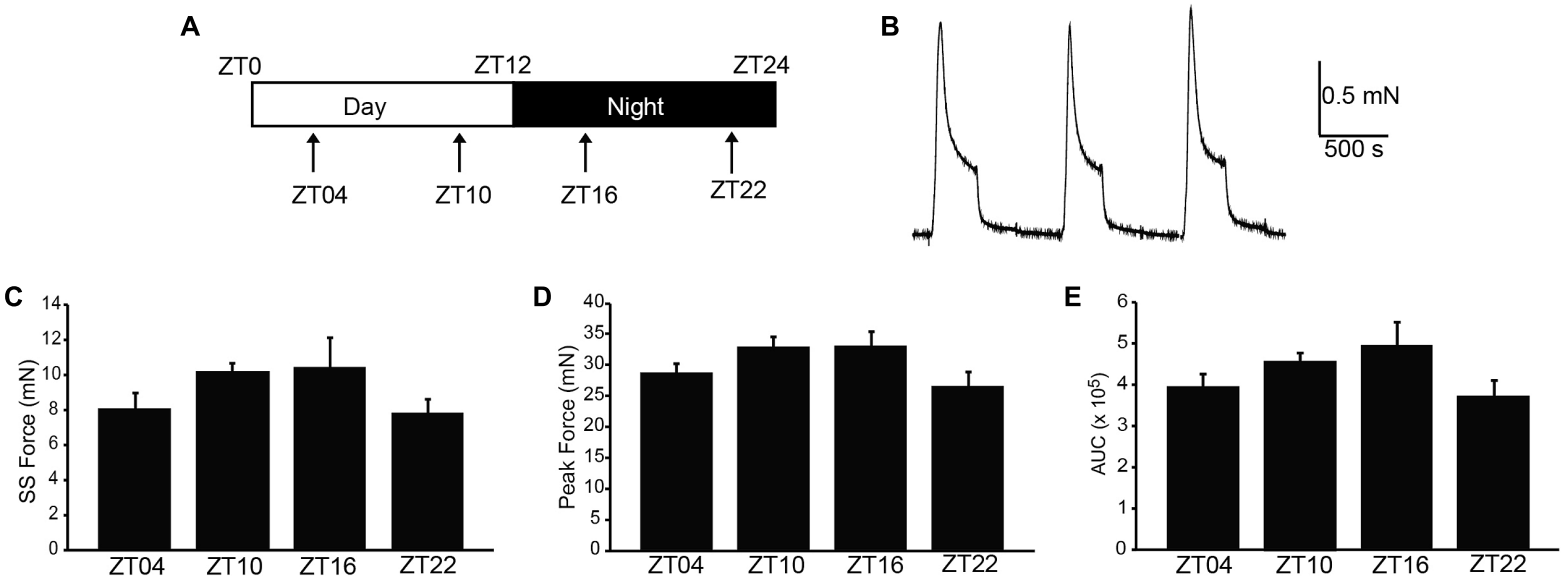
**High K^+^-induced excitation–contraction responses are similar across the circadian cycle in isolated UBSM strips. (A)** Schematic of the circadian cycle and four time points examined in this study. Mice are nocturnal, resting during the daytime light period, zeitgeber time (ZT) 0–12, and being active at night during the nighttime dark period, ZT12-24. **(B)** Representative 60 mM KCl-induced contractions. KCl was applied for 5 min, followed by a 10 min washout. Contraction values were obtained from the third KCl application. **(C)** Peak amplitude. **(D)** Steady-state amplitude. **(E)** Area under the curve (AUC). All data are mean ± SE, *n* = 5–6 mice (20–24 UBSM strips) per timepoint. For all comparisons, *p* > 0.05 (one-way ANOVA).

In nocturnal rodents, micturition frequency is higher during the night (active) period, compared to daytime. We hypothesized that strips of UBSM tissue harvested during the dark period would demonstrate stronger contractile activity than strips harvested during the day, when micturition frequency is low and the bladder relaxes to store urine (Herrera and Meredith, 2010; Negoro et al., 2012). Thus, to determine whether contractile properties of UBSM varied by time of day, isometric tension recordings were performed at ZT4, 10, 16, and 22 (**Figure 1A**). Isolated strips were denuded of the urothelium, but nerve terminals are retained in this prep, enabling both spontaneous and nerve-evoked contractions (Kullmann et al., 2014). UBSM strips were affixed to a solid support, and an initial stretch was applied (1.5 g). After the initial relaxation, 60 mM KCl was applied to induce depolarization of the muscle and elicit a maximal contraction (**Figure 1B**).

To characterize whether a daily rhythm was present contractile activity, the KCl-induced responses were compared across time points. No significant differences were found in the peak, steady-state, or integrated KCl-induced amplitudes between timepoints (**Figures 1C–E**). Application of higher initial tension (2.5 g) also did not reveal any significant difference in maximal KCl-induced amplitude (**Table 1**). These data suggest that the basic contractile apparatus does not undergo daily alterations that have a major consequence on function.

**Table 1:** Effect of time of day on UBSM contractility at two initial tensions.

	1.5 g	2.5 g
**Initial tension**
ssKCl-Induced amplitude	ns	ns
Phasic amplitude	*ZT4/ZT10	*ZT4/ZT10
Phasic frequency	ns	ns
EFS amplitude	ns	ns
Half-max frequency	ns	ns
**Paxilline**
Phasic amplitude	*ZT17	
Phasic frequency	ns	
EFS amplitude	ns	

For 1.5 g initial tension, *n*’s are reported in the previous figure legends. For 2.5 g initial tension, *n* = 8 animals (1–2 UBSM strips averaged per animal). ssKCl, steady-state KCl-induced amplitude. ns, no significant difference across timepoints (*p* > 0.05, one-way ANOVA). *p < 0.05 (one way ANOVA, and the indicated *post hoc* comparison (Bonferroni) was significant at *p* < 0.05.

**FIGURE 2:**
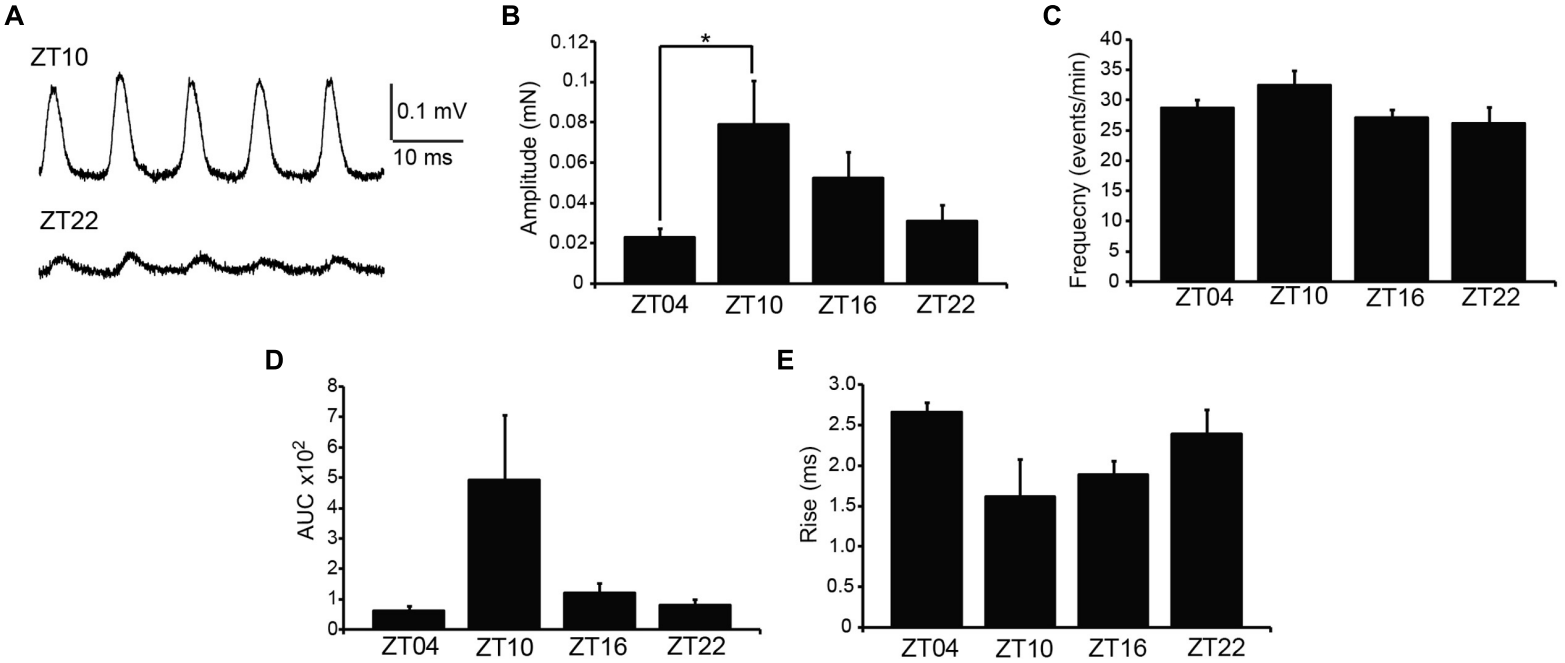
**Spontaneous (phasic) contractions are larger at ZT10. (A)** Representative phasic contractile activity at ZT10 and ZT22. **(B)** Phasic amplitude differs by time of day. *p* = 0.03 (one-way ANOVA), *Bonferroni *post hoc*, *p* < 0.04. **(C)** Phasic frequency is not different across time points, *p* = 0.17 (one-way ANOVA). **(D)** AUC. *p* = 0.03 (one-way ANOVA).** (E)** Rise time of phasic events. *p* = 0.08 (one-way ANOVA). All data are mean ± SE, *n* = 6–7 mice (10–12 UBSM strips) per timepoint.

Next, we addressed whether phasic activity in UBSM strips differed by time of day. Phasic contractions result from the spontaneous action potential activity of smooth muscle cells within the UBSM strip (Brading, 1997). Phasic contractions are proposed to be important in maintaining bladder tone, and reduction of phasic contractility is correlated with bladder relaxation to accommodate filling (Herrera et al., 2003; Kullmann et al., 2014). Greater than 80% of strips exhibited phasic contractions, similar to previous results on this mouse strain background (Herrera et al., 2003). There was no significant difference in the number of strips with phasic activity at each time point (*p* > 0.05, Fisher’s Exact test, *n*’s as indicated in **Figure 2** legend). These results show that phasic activity is generated throughout the daily cycle.

Urinary bladder smooth muscle strips isolated at ZT4 had the lowest phasic amplitudes (**Figures 2A,B**). By ZT10, phasic amplitude was fourfold greater than ZT4 (**Figure 2B**). The amplitudes decreased at ZT16 and ZT22 (**Figure 2B**). Fitting the data to a cosine function also established ZT10 as the peak contractile amplitude of the 24-hr rhythm (*p* = 0.01, Refinetti et al., 2007). Similarly, the integrated area of the phasic contraction was greater at ZT10 (**Figure 2D**). Although not significant, the time to peak contraction (rise time) was shorter on average at ZT10. Furthermore, in independent experiments, phasic activity from UBSM with a higher initial tension applied also showed a significant difference between ZT4 and ZT10 contractile amplitudes (**Table 1**). Taken together, these data suggest that a daily rhythm in phasic contractile amplitude is present in UBSM. In contrast, there was no significant difference in the frequency of phasic contractions across the daily cycle (**Figure 2C**; **Table 1**).

### NERVE-EVOKED CONTRACTILE ACTIVITY IN UBSM AT DIFFERENT TIMES OF DAY

**FIGURE 3:**
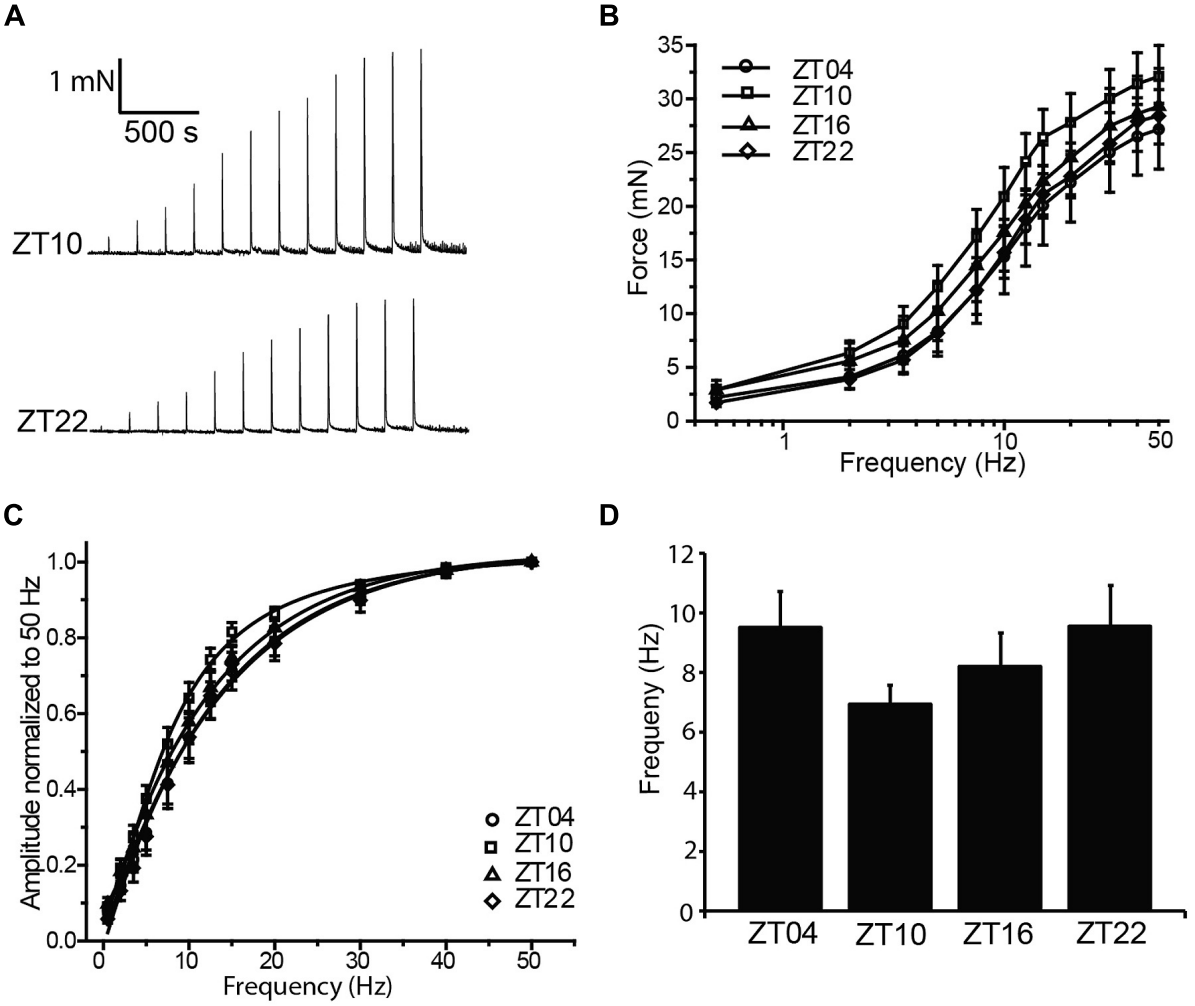
**Nerve-evoked (EFS) contractions are not different between timepoints. (A)** Representative EFS-evoked contractions at ZT10 and ZT22. **(B)** EFS-evoked contractions, elicited by 0–50 Hz stimulation, are not different between timepoints. **(C)** EFS-evoked amplitudes normalized to the maximal amplitude at 50 Hz. **(D)** Frequency of half-maximal activation, derived from fits of data in **(C)**, was not different between timepoints. All data are mean ± SE, *n* = 6 mice (12 UBSM strips) per timepoint. For all comparisons, *p* > 0.05 (one-way ANOVA).

Coordinated bladder contraction during micturition is controlled by the parasympathetic nerves encapsulated in the bladder wall (Andersson and Arner, 2004). To investigate diurnal differences in nerve-evoked contractile activity, nerve-mediated release of neurotransmitter was elicited by electrical field stimulation (EFS). Physiological frequencies from 0.5 to 50 Hz, mimicking the excitation that occurs during micturition *in vivo*, were applied to strips harvested at different times of day, and the peak contractile responses were measured (**Figure 3A**). In the presence of 1 μM tetrodotoxin, no contractile response could be elicited (*n* = 3), validating that the contractions in response to EFS at each frequency were entirely derived from nerve activity.

Increasing stimulation frequencies produced greater contractile force (**Figures 3A,B**). However, no significant differences in EFS-evoked contractions across time points were found (**Figure 3B**; **Table 1**). To reveal any frequency-dependent differences across timepoints, contraction amplitudes at each frequency were normalized to the maximal EFS-evoked response at 50 Hz (**Figure 3C**). While no significant differences were obtained, ZT10 showed a slight reduction in the frequency of half-maximal contraction (**Figure 3D**), suggesting a trend toward enhanced sensitivity to nerve-mediated stimulation at ZT10. Nevertheless, on the whole, no substantial differences were found that would provide clear evidence of a daily rhythm in nerve-mediated contraction of UBSM tissue.

### BK CHANNEL EXPRESSION AND FUNCTION IN UBSM AT DIFFERENT TIMES OF DAY

**FIGURE 4:**
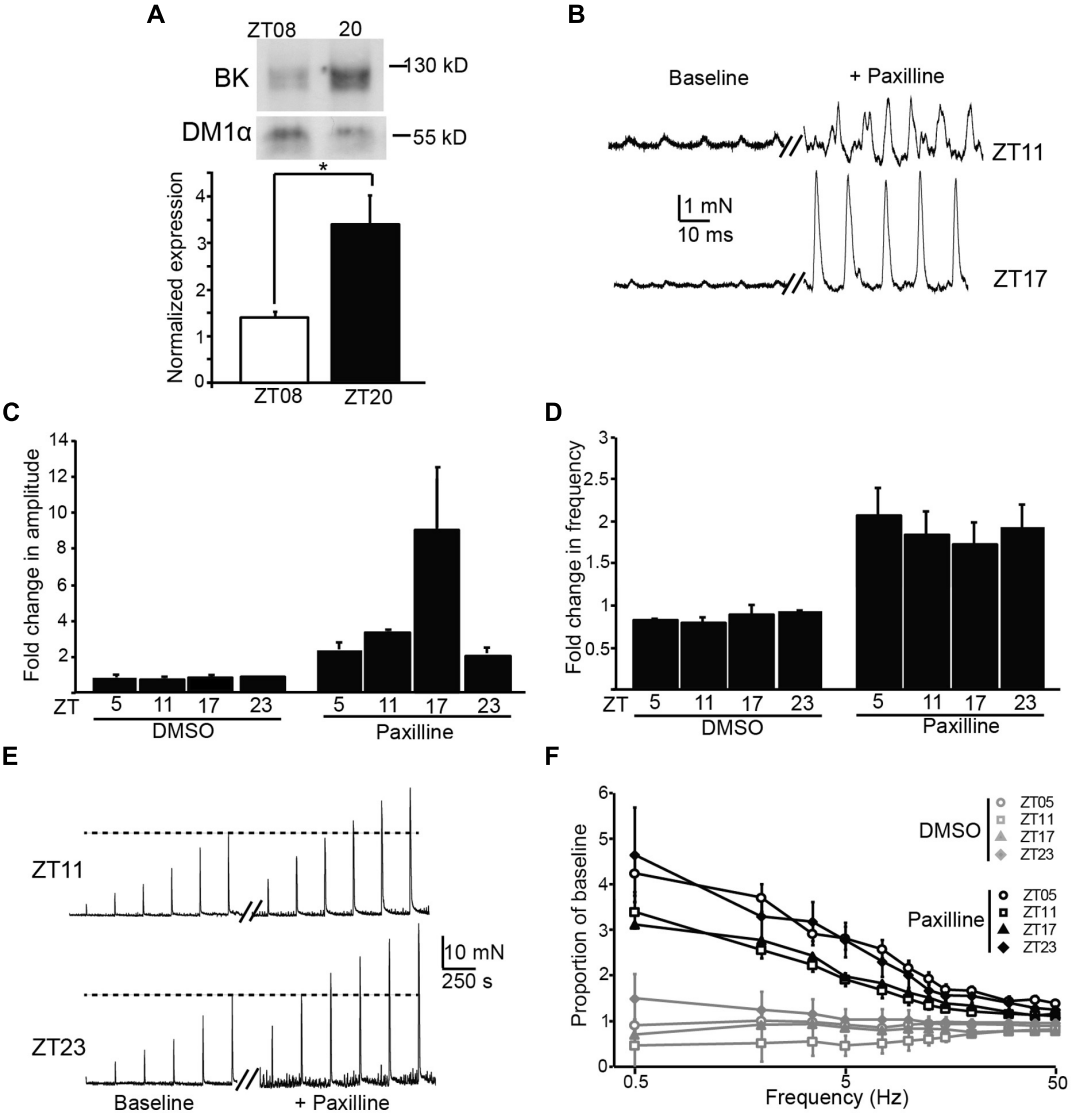
**Expression and functional impact of the BK channel on UBSM contractility at different times of day. (A)** Representative Western blot showing BK channel and DM1α protein expression at ZT8 and ZT20 (top). Average BK expression normalized to DM1α (bottom). ZT8 expression is significantly lower than ZT20 (*p* = 0.03, *t*-test, *n* = 4 mice per timepoint). **(B)** Representative phasic contractile activity at ZT11 and ZT17, at baseline and after Paxilline (PAX) application. **(C,D)** Fold-increase in phasic amplitude **(C)** and frequency **(D)** after PAX or DMSO (control) application. The effect of PAX and time were significant for phasic amplitude (factorial ANOVA, *p* = 10^-4^ and 0.01, respectively), but only the effect of PAX was significant on phasic frequency (*p* = 10^-8^). *n* = 3–6 mice (6–12 UBSM strips) per timepoint and condition. DMSO had no effect on either parameter. **(E)** Representative EFS-evoked contractile activity at ZT11 and ZT23, at baseline and after PAX application. **(F)** Increase in EFS-evoked amplitude after PAX or DMSO (control) as a proportion of baseline. The effect of PAX was significant (factorial ANOVA, *p* = 10^-5^), but the effect of time was not (*p* = 0.99). *n* = 5–6 mice (10–12 UBSM strips) per timepoint and condition. All data are mean ± SE.

BK channels are major regulators of UBSM excitability, and block or loss of BK channel activity in UBSM leads to increased phasic and EFS-evoked contractile amplitude and frequency (Meredith et al., 2004; Thorneloe et al., 2005). In addition, BK channels are also key regulators of the circadian rhythm in pacemaker excitability in the brain (Meredith et al., 2006; Kent and Meredith, 2008; Montgomery et al., 2013). To determine whether there was any evidence for BK channel involvement in the daily variation in UBSM phasic contractility, we first assessed the expression of BK from bladders harvested at ZT8 versus ZT20. BK expression was low at ZT8 (**Figure 4A**), similar to the time window with the highest phasic contractile amplitudes (**Figures 3A,B**). Conversely, BK expression was higher at ZT20, when phasic amplitudes were lower. The 2.3-fold increase in BK expression at ZT20 compared to ZT8 was similar to the difference in magnitude between the peak and trough of BK expression in the SCN circadian pacemaker (Meredith et al., 2006).

To determine the functional impact of blocking BK channels at different times of day, we applied a BK channel blocker, PAX, to UBSM strips and recorded phasic and EFS-evoked contractile responses. The results are plotted as the proportional change after PAX from baseline. We found an increase in both the phasic amplitude and frequency after application of PAX (**Figures 4B–D**), but not after application of DMSO (control). The effect of PAX to enhance phasic contractions is consistent with previous data showing the BK channel to be a critical suppressor of UBSM contractility (Meredith et al., 2004). The PAX-induced increase in phasic frequency did not vary by time of day (**Figure 4D**). However, the PAX-induced increase in phasic amplitude was highest at ZT17 (**Figures 4B,C**), parallel to the increased BK protein expression observed at ZT20 (**Figure 4A**). These data suggest that inhibition of BK channel activity has a limited diurnal effect on phasic contractile amplitude, and the time window of the enhanced effect of PAX is consistent with the phase of increased BK protein expression in bladder.

Application of PAX also resulted in an enhancement of EFS-evoked amplitudes (**Figures 4E,F**). The PAX-induced increase in EFS-evoked amplitude was frequency dependent, with a larger proportional increase at low compared to high frequencies. Nevertheless, the PAX-induced increase in EFS-evoked amplitudes was not found to significantly differ by time of day (**Figure 4F**). Thus the results obtained with PAX generally corroborate the pattern of diurnal changes observed in baseline contractility – i.e., an effect on phasic amplitude, but not phasic frequency or EFS-evoked contractions.

## DISCUSSION

Mice have recently been shown to express a bona fide circadian rhythm in micturition, and this rhythm has been proposed to rely on an intrinsic clock housed within UBSM (Negoro et al., 2012; Noh et al., 2014). This hypothesis predicts that strips of UBSM tissue harvested across circadian timepoints would demonstrate cyclic alterations in contractile properties. The central finding of this study was that acutely isolated UBSM tissue exhibits only a limited diurnal difference in contractile properties. We did not find significant evidence for rhythms in the output of the basic (KCl-induced) contractile apparatus in UBSM (**Figure 1**), or in nerve-evoked contractions (**Figure 3**). Instead, we found a single major difference in the amplitude, but not frequency, of phasic contractions (**Figure 2**). Notably, the observations were consistent across datasets from UBSM strips with two different initial tensions applied (**Table 1**). Taken together, these data did not show the expected co-variance of related parameters that would provide strong support for a diurnal rhythm in UBSM contractile properties. Furthermore, these data suggest the conclusion that UBSM possesses only limited intrinsic machinery for functional autonomous control of contractility.

Although limited, the time of day-dependent difference in phasic contraction identified here could potentially involve the activity of the BK K^+^ channel. The BK channel has been previously shown to regulate phasic contractions in UBSM (Meredith et al., 2004). The increased phasic amplitude at ZT10, compared to other timepoints, parallels the lower expression of BK protein in UBSM at ZT8. A reduction of BK expression at this time could facilitate the observed increase in phasic amplitude. Similarly, application of PAX, an inhibitor of BK channel activity, produced the largest effect on phasic amplitude at ZT17, near the timepoint of higher BK protein expression (ZT20, **Figure 4**). Although these data are suggestive of BK channel involvement in the daily difference in phasic activity, not all the results fit this hypothesis. For example, BK channel antagonists are also known to significantly enhance phasic frequency and EFS-evoked contractions (Meredith et al., 2004). Yet no significant time of day difference could be detected in these parameters at baseline or after BK inhibition with PAX. It is not clear how BK channel function would contribute selectively to suppressing phasic amplitude at ZT17, when PAX has a maximal effect, but not have an impact on phasic frequency or EFS-evoked contractions. Future studies that directly address the nature of excitation–contraction coupling at different times of day will be required to address this dilemma.

One question that remains outstanding is the functional significance of the diurnal rhythm in phasic amplitude. Phasic contractions are thought to be important for maintaining bladder tone, decreasing with bladder relaxation to accommodate filling (Herrera et al., 2003; Kullmann et al., 2014). The increase in phasic contractile amplitude at ZT10, a timepoint which occurs at the end of the rest (light) phase, may indicate the bladder is intrinsically programmed to switch out of a urine storage mode (light phase) to facilitate increased micturition when entering the active (dark) phase. Recordings of bladder capacity from rats in the day or night are consistent with this idea (Herrera and Meredith, 2010), but concomitant measurements of UBSM and bladder properties in the same animal model across timepoints will be required to correlate the precise phase relationship.

From a clinical perspective, understanding the underlying pathology of nocturia will require identifying the circadian mechanisms that are deranged in the pathophysiological state. Systemic disruption of the mechanism for encoding circadian rhythm, via mutation of *Cry1/Cry2* or *Per1/Per2* double knockout mice, alters both the circadian pattern of micturition and gene expression (Negoro et al., 2012; Noh et al., 2014). However, tissue-specific deletions will be necessary to parse out the relative contributions of central, renal, and peripheral clocks to the circadian rhythm in urodynamics. To date, the lower urinary tract has not been comprehensively investigated as a contributor to nocturia. However, the results reported here showing minimal diurnal differences in USBM contractility contrast with recent reports of robust circadian oscillations reported in cultured bladder cells expressing a *Per2*-luciferase reporter and clock gene expression in acutely harvested bladder tissue (Negoro et al., 2012). Our data suggest the possibility that these oscillations in gene expression may not drive intrinsic rhythms in UBSM contractile activity in a meaningful way.

The only other study to provide data directly addressing the presence of intrinsic rhythms in contractility found a circadian rhythm in muscarinic-stimulated UBSM contraction, but no clear rhythm in either nerve-evoked or direct muscle-stimulation evoked responses (Wu et al., 2014). Although this study differed methodologically from the data presented here, where Wu et al. (2014) cultured the bladder strips and applied dexamethasone to synchronize circadian rhythmicity, it could be interpreted as consistent with our data with respect to a lack of rhythmicity in EFS-evoked contractions. Taking these initial investigations together, the lack of a robust circadian rhythm in UBSM contractility in our study, and the emergence of a circadian difference only with muscarinic-stimulated UBSM contraction (Wu et al., 2014), underscores the importance of continued investigation of alternative mechanisms focusing on both descending outflow through autonomic control of the bladder, as well as the kidney and polyuria, in the treatment of nocturia.

## AUTHOR CONTRIBUTIONS

Rachel S. White, Betsir G. Zemen, Zulqarnain Khan, Andrea L. Meredith, and Gerald M. Herrera designed the experiments. Rachel S. White, Betsir G. Zemen, and Zulqarnain Khan performed the experiments. Jenna R. Montgomery assisted with data analysis, performed statistical analysis, and prepared figures. Andrea L. Meredith wrote the manuscript. All authors approved the final version of the manuscript.

## Conflict of Interest Statement

The authors declare that the research was conducted in the absence of any commercial or financial relationships that could be construed as a potential conflict of interest.
